# 3D Buried Utility Location Using A Marching-Cross-Section Algorithm for Multi-Sensor Data Fusion

**DOI:** 10.3390/s16111827

**Published:** 2016-11-02

**Authors:** Qingxu Dou, Lijun Wei, Derek R. Magee, Phil R. Atkins, David N. Chapman, Giulio Curioni, Kevin F. Goddard, Farzad Hayati, Hugo Jenks, Nicole Metje, Jennifer Muggleton, Steve R. Pennock, Emiliano Rustighi, Steven G. Swingler, Christopher D. F. Rogers, Anthony G. Cohn

**Affiliations:** 1 School of Computing, University of Leeds, Leeds LS2 9JT, UK; L.J.Wei@leeds.ac.uk (L.W.); D.R.Magee@leeds.ac.uk (D.R.M.); A.G.Cohn@leeds.ac.uk (A.G.C.); 2 School of Electronic, Electrical and Computing Engineering, University of Birmingham, Birmingham B15 2TT, UK; P.R.Atkins@bham.ac.uk (P.R.A.); F.Hayati@bham.ac.uk (F.H.); 3 School of Civil Engineering, University of Birmingham B15 2TT, UK; D.N.Chapman@bham.ac.uk (D.N.C.); Giulio.Curioni@gmail.com (G.C.); N.Metje@bham.ac.uk (N.M.); C.D.F.Rogers@bham.ac.uk (C.D.F.R.); 4 School of Electronics and Computer Science, University of Southampton, Southampton SO17 1BJ, UK; kfg@ecs.soton.ac.uk (K.F.G.); sgs@ecs.soton.ac.uk (S.G.S.); 5 School of Electronic and Electrical Engineering, University of Bath, Bath BA2 7AY, UK; C.H.J.Jenks@bath.ac.uk (H.J.); S.R.Pennock@bath.ac.uk (S.R.P.); 6 Institute of Sound and Vibration Research, University of Southampton, Southampton SO17 1BJ, UK; jmm@isvr.soton.ac.uk (J.M.); er@isvr.soton.ac.uk (E.R.)

**Keywords:** buried utility location, marching-cross-section algorithm, multi-sensor data fusion

## Abstract

We address the problem of accurately locating buried utility segments by fusing data from multiple sensors using a novel Marching-Cross-Section (MCS) algorithm. Five types of sensors are used in this work: Ground Penetrating Radar (GPR), Passive Magnetic Fields (PMF), Magnetic Gradiometer (MG), Low Frequency Electromagnetic Fields (LFEM) and Vibro-Acoustics (VA). As part of the MCS algorithm, a novel formulation of the extended Kalman Filter (EKF) is proposed for marching existing utility tracks from a scan cross-section (scs) to the next one; novel rules for initializing utilities based on hypothesized detections on the first scs and for associating predicted utility tracks with hypothesized detections in the following scss are introduced. Algorithms are proposed for generating virtual scan lines based on given hypothesized detections when different sensors do not share common scan lines, or when only the coordinates of the hypothesized detections are provided without any information of the actual survey scan lines. The performance of the proposed system is evaluated with both synthetic data and real data. The experimental results in this work demonstrate that the proposed MCS algorithm can locate multiple buried utility segments simultaneously, including both straight and curved utilities, and can separate intersecting segments. By using the probabilities of a hypothesized detection being a pipe or a cable together with its 3D coordinates, the MCS algorithm is able to discriminate a pipe and a cable close to each other. The MCS algorithm can be used for both post- and on-site processing. When it is used on site, the detected tracks on the current scs can help to determine the location and direction of the next scan line. The proposed “multi-utility multi-sensor” system has no limit to the number of buried utilities or the number of sensors, and the more sensor data used, the more buried utility segments can be detected with more accurate location and orientation.

## 1. Introduction

Most utility services, including electricity, water, gas and telecommunications, are distributed using buried pipes or via directly buried cables, and the majority of these buried utility infrastructures exist beneath roads. Millions of holes are dug every year in the whole world in highways and footpaths in order to maintain, repair, extend or replace the existing utility services [1]. The inaccurate location of buried pipes and cables results in far more excavations than necessary, thereby increasing the direct costs of maintenance to the service providers and causing enormous traffic delays. There is a strong need to accurately locate and identify the buried utilities in a local area before any excavation begins. Historically, locations of buried utilities are provided by record information held by utility companies. However, this information may be incomplete, inaccurate or not up-to-date, especially with regard to the depth information. Different geophysical sensors/techniques have been designed to locate buried utilities [2], such as pipe detection with vibro-acoustic methods [3,4,5], electrical cable detection with passive magnetic fields [6], buried asset detection with low-frequency electromagnetic sensors [7], buried target detection using Ground Penetrating Radar (GPR) [8,9] and water pipe detection using small sensors incorporated into the pipe [10]. Different sensors/techniques have their own advantages and limits for buried utility location in different environment conditions. For example, the Vibro-Acoustics (VA) of ground excitation works better for detecting assets under grass-covered areas than assets under tarmac, whereas on the other hand, GPR works better on tarmac than on grass-covered areas as the ground could be wetter under grass and, thus, have a higher conductivity, reducing the transmission of radar waves. Passive Magnetic Fields (PMF) are used to detect buried cables with electric current passively, and Low Frequency Electromagnetic Fields (LFEM) can be used to detect both pipes and cables [11]. If these multi-sensor data could be integrated appropriately, a more complete and accurate buried utility network could be reconstructed.

A key component of buried utility location is how to connect those individual hypothesized detections from different sensors to generate utility segments in 3D [11]. In practice, locations with high responses from a detecting sensor are marked on the ground surface with colour-coded paint, then connected by hand by experts to estimate the buried utility lines. Some methods have been proposed to automate this procedure [12,13,14]. In [12], the authors use a dynamic Bayesian network to integrate VA data and GPR data to find the buried pipe location and depth. In [13], the authors proposed to use GPS, GPR and GIS for mapping underground utilities. In [14], the individual hypothesized detections from a group of parallel GPR scans are used to determine the approximate location and direction of a buried pipe segment by assuming that there is only one utility in the surveyed area and the utility is straight. However there might be multiple utilities; the utilities may not be straight; they may be very close to each other or even intersect at some points. In addition, if multiple groups of sensor data are captured in the same area with the scan lines of each group going in different directions, how to make use of all of this information in a general framework to find all potential buried utilities is an open question. When multiple sensors are used, one utility may be detected by multiple sensors at the same point, and different sensors may detect different utility segments; how can we use this repeated and complementary information to solve the “multi-target multi-observation” problem? These open questions relating to multi-sensor data fusion for buried utility location are tackled in this work.

In this paper, we propose a Marching-Cross-Sections (MCS) algorithm to automatically integrate the individual hypothesized detections from multiple sensors to locate the 3D buried utility segments in a surveyed area. The proposed “multi-utility multi-sensor” system has no limit to the number of buried utilities or the number of sensors. By discretizing the 3D space under the surveyed area with scan cross-sections (scss, the definitions of scan cross-section (scs) and scan line are given in Section 2.2), the MCS algorithm goes forward from one scs to the next to locate utility segments based on the given or extracted hypothesized detections (a detailed explanation of individual hypothesized detection is given in Section 3.1). There are five main components of the MCS algorithm: utility track initialization, track marching, data association, track updating and track management. Hypothesized detections from different sensors are grouped based on their Mahalanobis distances and used to initialize utility tracks, including the utility locations, orientations and probabilities to be a pipe or a cable on the first scs. After the utility tracks are initialized, they are predicted forward to the next scs along the estimated longitudinal direction of the utilities. Rules are defined for associating corresponding hypothesized detections to existing utility tracks. In the tracks updating stage, all hypothesized detections associated with a certain utility track are used to update this utility sequentially using a Kalman Filter (KF) [15,16,17,18,19]. Finally, several rules are defined to manage utility tracks to keep all potentially correct ones and to reject invalid ones. This step includes utility merging, utility splitting and utility pruning. If in a survey, different sensors share common scan lines, then the scss are generated from the actual survey scan lines; otherwise, a group of virtual scan lines are generated automatically based on the extracted hypothesized detections of utilities with the algorithms proposed in this paper. Though the segment between two adjacent scss is assumed to be locally linear, the proposed MCS algorithm can locate curved utilities accurately. Besides being used for post-processing of data captured by multiple sensors, the proposed algorithm can also be used for on-site application when a survey is on going. In this way, the detected tracks till the current scs can help to determine the location and direction of the next survey scan line.

The rest of this paper is organised as follows: the sensors used in this work and the related data acquisition and interpretation approaches are described in Section 2; then, the proposed MCS algorithm using actual survey scan lines and using virtual scan lines is presented respectively in Section 3 and Section 4; after that, experimental results on both synthetic and real data are shown and analysed in Section 5; and finally, conclusions are drawn in Section 6.

## 2. Sensor Data Interpretation and Registration

In this section, we present the sensors used for buried utility location in this work and explain how the sensor data are interpreted and spatially registered in subsurface surveys.

### 2.1. Sensors and Sensor Data Interpretation

**Figure 1:**
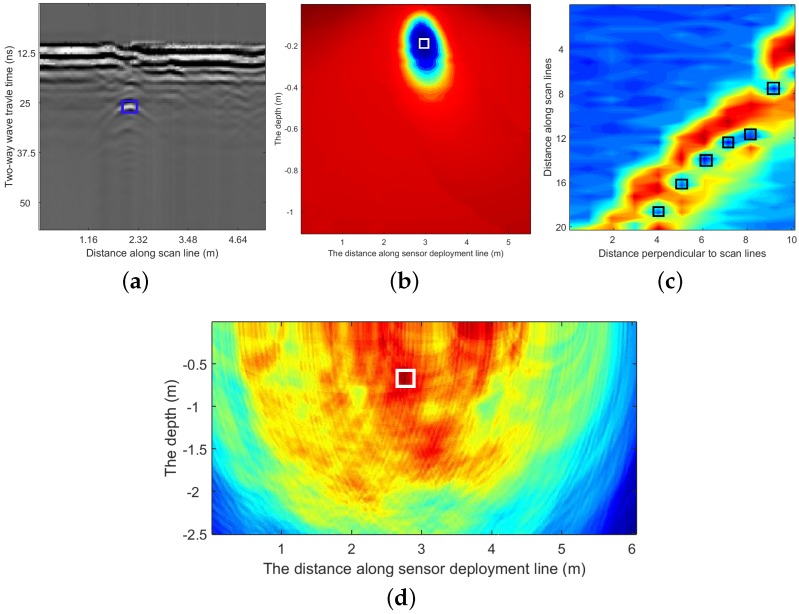
Different sensor images with extracted hypothesized detections of utilities marked by squares. (**a**) A Ground Penetrating Radar (GPR) image; (**b**) a Passive Magnetic Fields (PMF) image; (**c**) a Magnetic Gradiometer (MG) image; (**d**) a Vibro-Acoustic (VA) image. In (a,b,d), columns correspond to horizontal positions, and rows are related to depths. (c) is measured within a region on the ground surface.

In this work, five types of geophysical sensors comprising Ground Penetrating Radar (GPR), Passive Magnetic Fields (PMF), Magnetic Gradiometer (MG), Low-Frequency Electromagnetic Fields (LFEM) and Vibro Acoustics (VA) are used together to locate the buried utilities. Data captured by different sensors are processed individually to extract the hypothesized detections of buried utilities. Some examples of sensor data and their hypothesized detections are shown in Figure 1. The techniques for sensor data interpretation are briefly introduced in this section, for more details, please refer to [7,11,20,21,22].

- GPR is one of the most used techniques to locate both metallic and non-metallic buried utilities. It is an active instrument that transmits electromagnetic waves into the ground and collects the reflected signals from subsurface structures. By pushing a GPR sensor along a scan line, a GPR image is captured, as seen in Figure 1a. The vector of reflections measured at one certain position for different answering times (travel time) is called an A-scan. A sequence of consecutive A-scans composes a B-scan, which can be considered as a matrix of reflection intensities with rows corresponding to the answering time and columns corresponding to horizontal positions on the scan line. GPR data can be processed and interpreted manually by experts or using (semi-) automatic algorithms to find buried utilities represented by hyperbolic signatures in the GPR images [8,20,23,24]. In this paper, the hypothesized detections from GPR images are annotated manually; an example is shown in Figure 1a.
- PMF utilizes the oscillating magnetic field created by the flow of current within a buried cable to locate it [25]. As the current flow within a power cable can also induce currents within neighbouring utility pipelines or ducts made from conducting materials, PMF is also capable of detecting the magnetic fields indirectly generated from the nearby metallic objects. However, as a passive sensor, it can only detect the utilities with a flow of current; non-conductive materials, such as plastic pipes, cannot be detected by this technique. The PMF sensor used in this work is made of an array of 27 coils mounted on a frame to measure the magnetic field above buried cables. The hypothesized locations of buried cables are estimated by minimizing the error between the measured magnetic field values and those predicted by a simple numerical model of one or more cables [21,25,26]. The results are presented as an error map (an example is shown in Figure 1b); the lowest error is related to the most likely location for the cable.
- The MG sensor used in this work is composed of four coils evenly spaced vertically on a plastic pole. By analysing the changes of the signals produced by moving the coils, the position of the buried cable can be estimated [27,28]. Concretely, the differences of the magnitude values of the captured magnetic fields by different coils are calculated, and the local valleys of the differences along the survey line are automatically selected, which are considered as hypothesized detections from this technique. An example is shown in Figure 1c.
- LFEM is a method of measuring anomalies in the electrical resistivity of the ground using non-contact methods. In this work, a sinusoidal alternating current is injected into the ground, and the sensed voltage is measured on two capacitively coupled plates moved along the surface. The ratio of voltage to current is proportional to the apparent resistivity of the ground. Any materials that present a contrast in electrical properties to the soil have the potential to be detected by this technique. The measurements are repeated on a regular grid, and the resulting image can reveal the underground infrastructure [7].
- VA-based techniques mechanically excite one part of the buried utility (via a manhole or valve) or the ground in a controlled way and measure the received response(s) at some remote location(s) on the ground surface using an array of geophones. By analysing the nature of the measured response(s) at the surface, the location of the buried pipe(s) can then be inferred [4]. These techniques are capable of detecting different types of pipes, and they work well in both dry and saturated areas, although it may not be suitable for detecting cables. In this work, the ground surface is excited, and the subsequent reflections arriving at multiple geophones are analysed to estimate the possible locations of buried pipes. The cross-correlation functions between the measured ground velocities and a reference measurement adjacent to the excitation are used to generate a cross-sectional image of the ground using a time domain stacking approach; then, local maxima are extracted from this image and used as the hypothesized detections [4,5,22]. An example of a cross-sectional stacking image is given in Figure 1d, in which the dark red region identifies the most possible locations of the pipe.

Based on the above description of the different sensors, it can be concluded that either the sensors are complementary to each other in some way or they can be used to verify each other. For example, GPR works better on tarmac than on grass-covered areas since electrically-conductive ground conditions cause significant attenuation losses of electromagnetic signals, resulting in shallow penetration depths for GPR. On the other hand, VA for ground excitation works better in grass-covered areas than on tarmac because the geophones used as receivers for VA can be inserted into the ground of the grass-covered area, and then, better signals can be captured. It may be difficult for GPR to detect thin cables, but PMF is less sensitive to the size of the cables as long as there is electrical current in the cable and the go and return cores in the cable have a reasonable distance from each other. By combining the results from multiple sensors, a more accurate or more complete buried utility network can be reconstructed.

### 2.2. Data Registration

**Figure 2:**
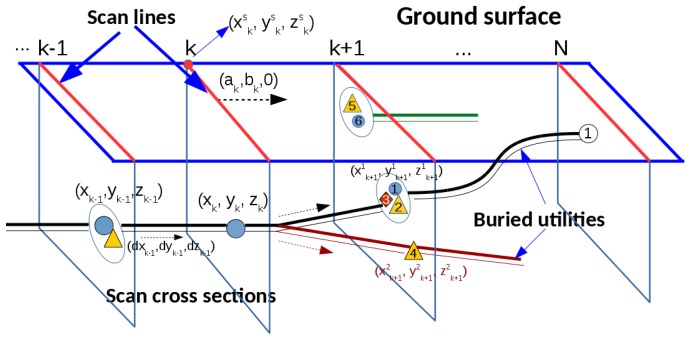
A schematic of the configuration of scan cross-sections. In this figure, $\left(a_{k},b_{k},0\right)$ is the normal vector of the *k*-th scan cross-section; $\left(x_{k}^{c},y_{k}^{c},z_{k}^{c}\right)$ are the coordinates of the start point of the *k*-th scan line; $\left(x_{k},y_{k},z_{k}\right)$ are the coordinates of a hypothesized detection on the *k*-th scan cross-section; and $\left(dx_{k},dy_{k},dz_{k}\right)$ represents the longitudinal direction of the related utility at the hypothesized detection.

In the subsurface survey, two GPRs, a PMF, a MG, a LFEM and a VA (ground excitation), are used. All of the sensor data are recorded and tied-in using marked grids in order to put all of the hypothesized detections of utilities in the same coordinate frame to facilitate data fusion. The geo-measurement with respect to the spatial coordinate frame is done by a `total station theodolite’. The line segment on a ground surface along which a sensor is applied is called a scan line, as depicted in Figure 2. A scan line can be specified by its start point and end point or by its start point, its direction and its length. A section below a scan line and perpendicular to the ground surface, which is assumed to be flat in this work, to a certain depth (2 m for example) is called a scan cross-section (scs). Examples can be seen in Figure 2 below the scan lines. Given the 3D coordinates of the start point of a scan line and another point on the same scan line, the normal vector of the related scs can be computed. The start point of a scan line and the normal vector of the related scs will be used in the formulation of the MCS algorithm described in the following sections.

## 3. Buried Utility Location with an MCS Algorithm

In this section, the MCS algorithm for locating buried utilities with data from multiple sensors is presented. This algorithm includes five key components: utility track initialization, track marching, data association, track updating and track management. Some assumptions for applying the MCS algorithm and the five components of the algorithm are described one by one in the following sections.

### 3.1. Assumptions

#### 3.1.1. Representation of Hypothesized Detections

In this work, the buried utilities are divided into two general groups, i.e., pipes and cables. At first, for a hypothesized detection from the data of a known sensor on a specific scs, based on the geo-measurement of the scs in the global coordinate system and the intrinsic parameters of the sensor, its 3D location can be computed. Besides the geo-measurement provided by the sensors, as different sensors usually have different capabilities to detect pipes or cables, the sensors can also provide the possible types of the located utilities based on their intrinsic characteristics; for example, if a sensor is designed mainly for detecting pipes and then a hypothesized detection from its data is believed to have a higher probability of being a response from a pipe than from a cable when nothing about the configuration of the buried utilities is known and vice versa.

Based on the above discussion, a hypothesized detection from a known sensor can be represented by: {x,y,z,pp,pc}, with *x*, *y*, *z* the 3D coordinates of the detection in the global frame and pp and pc the probabilities of the detection being a response from a pipe or from a cable, respectively. For example, a PMF sensor is designed to detect cables with electric current. Current may also be induced in the neighbouring metal pipes by the cables, but as the current generated in the metal pipe is usually weak, it is rarely detected. Therefore, for a PMF sensor, the value of pc is relatively large compared with the value of pp. Usually pp+pc<1; this is because, besides pipes and cables, the hypothesized detections maybe from other objects.

#### 3.1.2. The Uncertainty of Hypothesized Detection

As multiple hypothesized detections may be extracted on an scs from the data of a specific sensor, let $\left\{V_{k}^{i,1},\cdots ,V_{k}^{i,m}\cdots ,V_{k}^{i,N_{i}}\right\}$ be the set of $N_{i}$ hypothesized detections by the *i*-th sensor on the *k*-th scan cross-section $scs_{k}$, with corresponding uncertainties $\left\{R_{k}^{i,1},\cdots ,R_{k}^{i,m},\cdots ,R_{k}^{i,N_{i}}\right\}$, $m\in [1,N_{i}]$, and *i* satisfies i∈[1,S] with *S* the number of sensors.

**Figure 3:**
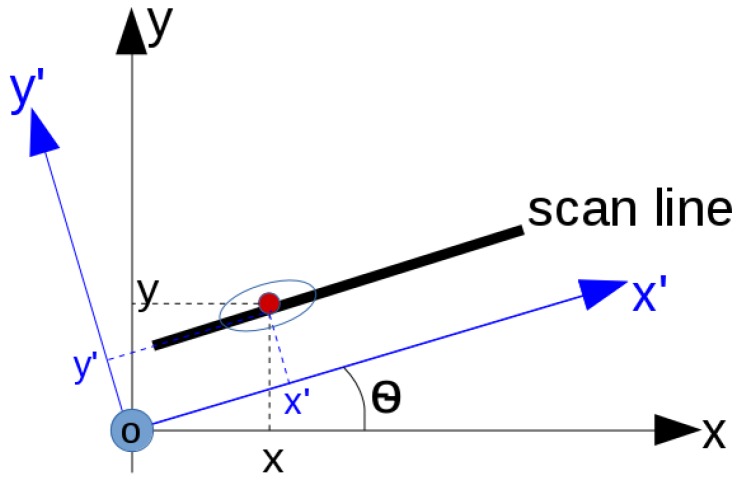
A local coordinate frame $x^{\prime }$-*o*-$y^{\prime }$ with respect to a scan cross-section and a global coordinate frame *x*-*o*-*y*.

The uncertainty of a hypothesized detection is related to several factors, such as the system noise of the sensor, the depth of the related hypothesized detection and the accuracy of the geo-positioning of the scan lines. In Figure 3, it can be seen that a given scan line is parallel to the $x^{\prime }$-axis, and in three-dimensional space, the related scs is parallel to the $x^{\prime }$-*o*-$z^{\prime }$ plane. If a hypothesized detection is extracted on this scs, its $\left(x^{\prime },y^{\prime }\right)$ coordinates are independent to each other since its $y^{\prime }$ coordinate is only decided by the $y^{\prime }$ coordinate of the start point, and its $x^{\prime }$ coordinate is decided by both the $x^{\prime }$ coordinate of the start point of the scan line and the horizontal distance of the hypothesized detection along the scan line. Based on the understanding of the sensors involved in this work, as the signal strength attenuates gradually with the depth increasing, the uncertainty of an extracted hypothesized detection will also increase. Therefore, for a hypothesized detection $\left(x^{\prime },y^{\prime },z^{\prime },pp,pc\right)$ on a scan line parallel to the $x^{\prime }$-axis, the uncertainty matrix of the measurement noise can be given as follows:

(1) $$R^{\prime }=\left(\begin{array}{ccccc} \omega_{x^{\prime }}^{2} & 0 & 0 & c & 0\\ 0 & \omega_{y^{\prime }}^{2} & 0 & 0 & 0\\ 0 & 0 & {\left(\omega_{z^{\prime }}*z^{\prime }\right)}^{2} & 0 & 0\\ 0 & 0 & 0 & \omega_{pp}^{2} & \omega_{pp}*\omega_{pc}\\ 0 & 0 & 0 & \omega_{pp}*\omega_{pc} & \omega_{pc}^{2} \end{array}\right)$$

In the above expression, the uncertainty along the scan line is coded by $\omega_{x^{\prime }}$, and the uncertainty along $y^{\prime }$-axis is coded by $\omega_{y^{\prime }}$. We expect that the geo-measurement of the start point of the scan lines is more accurate than that of the horizontal distance of the hypothesized detection, so the value of $\omega_{y^{\prime }}$ should be much smaller than that of $\omega_{x^{\prime }}$. The uncertainty of depth is related to the depth itself with a ratio $\omega_{z^{\prime }}$. In our experiments, the values of theses parameters are set according to the understanding of the related sensors, for example for a GPR hypothesized detection, $\omega_{x^{\prime }}=0.2,\;\omega_{y^{\prime }}=0.05,\;\omega_{z^{\prime }}=0.1$.

With respect to the *x*-*o*-*y* coordinate system, when the scan line is not parallel to the *x*-axis, the *x* and *y* coordinates of a hypothesized detection on the scan cross-section are not independent of each other in the 3D frame. Suppose the angle between the scan line and the *x*-axis is *θ*, θ∈(0,π) (Figure 3); if the coordinate system is rotated around the *z*-axis for an angle of *θ* anticlockwise to the $x^{\prime }$-axis, then the *x*-axis is parallel to the scan line. The relationship of the coordinates of the hypothesized detection in the 3D global frame (x,y,z) and in the rotated coordinate frame $\left(x^{\prime },y^{\prime },z^{\prime }\right)$ is written as follows:

(2) $$\left(\begin{array}{c} x\\ y\\ z \end{array}\right)=\left(\begin{array}{ccc} cos\theta & -sin\theta & 0\\ sin\theta & cos\theta & 0\\ 0 & 0 & 1 \end{array}\right)\left(\begin{array}{c} x^{\prime }\\ y^{\prime }\\ z^{\prime } \end{array}\right)$$

In this situation, the covariance matrix R of the measurement noise in the global coordinate frame is $R={\mathrm{AR}}^{\prime }A^{T}$, where $R^{\prime }$ is given by Equation (1) and A is given as follows:

(3) $$A=\left(\begin{array}{ccccc} cos\theta & -sin\theta & 0 & 0 & 0\\ sin\theta & cos\theta & 0 & 0 & 0\\ 0 & 0 & 1 & 0 & 0\\ 0 & 0 & 0 & 1 & 0\\ 0 & 0 & 0 & 0 & 1 \end{array}\right)$$

In this work, scan lines in the same survey group do not intersect in the surveyed area and can be thought as parallel to each other approximately. Scan lines in different groups can go in different directions and can intersect each other. A sensor can do multiple surveys along different groups of scan lines, and different sensors may not share the same scan lines.

For the description of the MCS algorithm in the following sections (Section 3.2, Section 3.3, Section 3.4, Section 3.5 and Section 3.6), we assume that all of the sensor data share the same group of scan lines. As for the situations where sensors use different groups of scan lines or no scan line information is provided for some sensors, a variant of the MCS algorithm is described in Section 4.

### 3.2. Initialization of Utility Tracks

Let $sl_{1},sl_{2},\cdots ,sl_{N}$ denote a group of parallel scan lines as shown in Figure 2; as the proposed MCS algorithm needs to be applied sequentially to a group of scan lines, it can be applied from $sl_{1}$ to $sl_{N}$ or from $sl_{N}$ to $sl_{1}$, which determines the direction in which the algorithm proceeds (called the marching direction in this work). To obtain a more complete or more accurate location of utility segments, the MCS algorithm is applied in both marching directions, and then, the results are merged into the final result as described in Section 3.6.

For a selected marching direction, the locations and orientations of the potential utilities are initialized with the extracted hypothesized detections on the first scs where some hypothesized detections are found. To initialize a utility track, if the utility is detected by multiple sensors, it should be initialized by fusing all of the corresponding hypothesized detections related to it. From the nature of the sensors, we have the prior information that two hypothesized detections from the same sensor on an scs are believed to be the responses from two different objects. Therefore, only hypothesized detections from different sensors should be used to initialise a utility track.

On a certain scs, a combination of hypothesized detections containing one hypothesized detection from each sensor, which has hypothesized detections on this scs, is called a maximum combination. For example, $\left\{V_{1}^{1,1},V_{1}^{2,1},\cdots ,V_{1}^{S,1}\right\}$ represents a maximum combination from *S* different sensors on the first cross-section, with corresponding covariances$\left\{R_{1}^{1,1},R_{1}^{2,1},\cdots ,R_{1}^{S,1}\right\}$ as defined in Section 3.1. The initialization procedure with respect to maximum combinations is given as follows:

1. Within each maximum combination, in order to take the uncertainties of hypothesized detection into account, the Mahalanobis distances $md_{ij}$ (i∈[1,S], j∈[1,S] and i≠j) between each pair of hypothesized detections in this combination are computed with the prior uncertainties of hypothesized detections defined in Equation (1) to Equation (3). Then,
  - if none of the Mahalanobis distances are less than a predefined threshold, this means no pair of the hypothesized detections is believed to come from the same utility. If so, no fusion will be done in this maximum combination;
  - if there are some Mahalanobis distances less than the threshold, the agglomerative clustering method [29] is employed to merge associated hypothesized detections using the Mahalanobis distance metric. The pair of hypothesized detections ($V_{1}^{i,1},V_{1}^{j,1}$) with the minimum Mahalanobis distance value are merged using a maximum likelihood formulation. The merged hypothesized detection $V_{1}^{\left(i,1;j,1\right)}$ and its uncertainty $R_{1}^{\left(i,1;j,1\right)}$ are calculated as follows:
    (4) $$R_{1}^{\left(i,1;j,1\right)}={\left({\left(R_{1}^{i,1}\right)}^{-1}+{\left(R_{1}^{j,1}\right)}^{-1}\right)}^{-1}$$
    (5) $$V_{1}^{\left(i,1;j,1\right)}=R_{1}^{i,1;j,1}\left({\left(R_{1}^{i,1}\right)}^{-1}V_{1}^{i,1}+{\left(R_{1}^{j,1}\right)}^{-1}V_{1}^{j,1}\right))$$
  - The merged hypothesized detection and the rest of the hypothesized detections in the original combination form a new combination $\left\{V_{1}^{\left(i,1;j,1\right)},\cdots ,V_{1}^{S,1}\right\}$. The Mahalanobis distances with respect to this new combination are computed and compared to the threshold. If the minimum Mahalanobis distance is less than the threshold, a further fusion will be done on the related pair and a new combination generated. This procedure continues until no Mahalanobis distance is less than the threshold. At this stage, the fusion results and the hypothesized detection used to do the fusions are recorded.
2. After going through all of the maximum combinations with the above procedure, two types of merged results will be specially treated:
  - some fusions can be repeated multiple times. For example, if a fusion with two hypothesized detections $V_{1}^{1,1}$ and $V_{1}^{2,1}$ is recorded with respect to a maximum combination, it may be met again in a later maximum combination. Therefore, once a fusion is recorded, the repeated ones will not be recorded any more.
  - Some fusions may be expanded from a recorded fusion. For example, a fusion is based on hypothesized detections $\left\{V_{1}^{1,1},V_{1}^{2,1},V_{1}^{3,1}\right\}$ and is recorded in the list, and later, a fusion based on $\left\{V_{1}^{1,1},V_{1}^{2,1},V_{1}^{3,1},V_{1}^{4,1}\right\}$ is found. In this situation, the latter one is regarded as an expansion from the previous one, and the one with fewer hypothesized detections is removed.
3. Finally, each recorded result of fusion is regarded as a utility and used to initialize a utility track. If a hypothesized detection is never used to initialize any utility track with others, it will initialize a track by itself.

There are several ways to initialize the utility orientations. For example, the initial orientation of a utility can be estimated from a manhole inspection if a survey is started close to a manhole or estimated based on statutory records if related statutory records are available. In this work, without any information of the manholes and the statutory records, the utility orientation is initialized in the direction perpendicular to the related scs. Then the initialized utility can be represented as: {x,y,z,pp,pc,dx,dy,dz}, called the state vector of the utility at location (x,y,z), with {dx,dy,dz} the estimated longitudinal direction of the utility at this location.

### 3.3. Marching of Utility Tracks

For a buried utility, if two adjacent scss are close enough (0.5 m for example), the utility segment between these two scss can be approximated with a linear segment. Suppose a utility is tracked to the (k−1)-th scan cross-section $scs_{k-1}$, as depicted in Figure 2, and the state vector of this utility on $scs_{k-1}$ is represented as $X_{k-1}={\left(x_{k-1},y_{k-1},z_{k-1},pp_{k-1},pc_{k-1},dx_{k-1},dy_{k-1},dz_{k-1}\right)}^{T}$. With the local linear assumption, the state vector of this utility on the *k*-th scan cross-section $scs_{k}$ can be predicted as follows:

(6) $$\left\{\begin{array}{ccc} x_{k|k-1} & = & x_{k-1}+l_{k}*dx_{k-1},\\ y_{k|k-1} & = & y_{k-1}+l_{k}*dy_{k-1},\\ z_{k|k-1} & = & z_{k-1}+l_{k}*dz_{k-1},\\ {pp}_{k|k-1} & = & pp_{k-1},\\ {pc}_{k|k-1} & = & pc_{k-1},\\ {dx}_{k|k-1} & = & dx_{k-1},\\ {dy}_{k|k-1} & = & dy_{k-1},\\ {dz}_{k|k-1} & = & dz_{k-1} \end{array}\right.$$

where:

(7) $$l_{k}=\frac{a_{k}\cdot \left(x_{k}^{s}-x_{k-1}\right)+b_{k}\cdot \left(y_{k}^{s}-y_{k-1}\right)}{a_{k}\cdot dx_{k-1}+b_{k}\cdot dy_{k-1}}$$

and $\left(x_{k}^{s},y_{k}^{s},z_{k}^{s}\right)$ denotes the start point of the *k*-th scan line and $\left(a_{k},b_{k},0\right)$ represents the normal vector of $scs_{k}$. The derivation of Equation (7) is given in Appendix A.

With the above procedure, a tracked utility can march from the current scs to the next one. Usually, it is assumed that the above process is subject to noise, and in this work, it is assumed as Gaussian. Therefore, the above procedure can be represented with a more compact form:

(8) $$\begin{array}{ccc} X_{k|k-1} & = & F_{k}X_{k-1}+q_{k} \end{array}$$

where $X_{k|k-1}=(x_{k|k-1},y_{k|k-1},z_{k|k-1},pp_{k|k-1},pc_{k|k-1},$ $dx_{k|k-1},dy_{k|k-1},dz_{k|k-1}{)}^{T}$ is the predicted state of a utility track on $scs_{k}$ and $q_{k}∽N\left(0,Q_{k}\right)$ is the Gaussian process noise.

The state transition function $F_{k}$ can be defined as follows:

(9) $$F_{k}=\left(\begin{array}{cccccccc} 1 & 0 & 0 & 0 & 0 & l_{k} & 0 & 0\\ \;0 & 1 & 0 & 0 & 0 & 0 & l_{k} & 0\\ 0 & 0 & 1 & 0 & 0 & 0 & 0 & l_{k}\\ 0 & 0 & 0 & 1 & 0 & 0 & 0 & 0\\ 0 & 0 & 0 & 0 & 1 & 0 & 0 & 0\\ 0 & 0 & 0 & 0 & 0 & 1 & 0 & 0\\ 0 & 0 & 0 & 0 & 0 & 0 & 1 & 0\\ 0 & 0 & 0 & 0 & 0 & 0 & 0 & 1 \end{array}\right)$$

where $l_{k}$ is defined in Equation (7).

The covariance of the predicted state vector $P_{k|k-1}$ on $scs_{k}$ is calculated with the Jacobian matrix $J_{k}\left(X\right)$ of $F_{k}$ as follows:

(10) $$\begin{array}{ccc} P_{k|k-1} & = & J_{k}\left(X\right)P_{k-1}J_{k}{\left(X\right)}^{T}+Q_{k} \end{array}$$

where $P_{k-1}$ is the covariance of the utility on $scs_{k-1}$; the Jacobian matrix $J_{k}\left(X\right)$ of $F_{k}$ on $scs_{k}$ is:

(11) $$J_{k}\left(X\right)=\left(\begin{array}{ccccccccc} u_{x} & -v_{x} & 0 & 0 & 0 & u_{x}\cdot l_{k} & -v_{x}\cdot l_{k} & 0\\ -u_{y} & v_{y} & 0 & 0 & 0 & -u_{y}\cdot l_{k} & v_{y}\cdot l_{k} & 0\\ -u_{z} & -v_{z} & 1 & 0 & 0 & -u_{z}\cdot l_{k} & -v_{z}\cdot l_{k} & l_{k}\\ 0 & 0 & 0 & 1 & 0 & 0 & 0 & 0 & \\ 0 & 0 & 0 & 0 & 1 & 0 & 0 & 0 & \\ 0 & 0 & 0 & 0 & 0 & 1 & 0 & 0\\ 0 & 0 & 0 & 0 & 0 & 0 & 1 & 0\\ 0 & 0 & 0 & 0 & 0 & 0 & 0 & 1 \end{array}\right)$$

where $u_{x}=\frac{b_{k}\cdot dy_{k-1}}{deno}$, $u_{y}=\frac{a_{k}\cdot dy_{k-1}}{deno}$, $u_{z}=\frac{a_{k}\cdot dz_{k-1}}{deno}$, $v_{x}=\frac{b_{k}\cdot dx_{k-1}}{deno}$, $v_{y}=\frac{a_{k}\cdot dx_{k-1}}{deno}$, $v_{z}=\frac{b_{k}\cdot dz_{k-1}}{deno}$ and $deno=a_{k}\cdot dx_{k-1}+b_{k}\cdot dy_{k-1}$.

### 3.4. Data Association

The utility states predicted from $scs_{k-1}$ to $scs_{k}$ are denoted as $X_{k|k-1}^{t}$, with $t\in [1,N_{u}]$ and $N_{u}$ the total number of predicted tracks as explained in Section 3.3. The $N_{u}$ predicted states are considered as hypothesized detections from a virtual sensor and are associated with the extracted hypothesized detections from real sensors on $scs_{k}$. With the new group of sensors (real sensors and the virtual one), the maximum combinations of hypothesized detections are generated the same way as in the initialization step. In each maximum combination, there is one and only one predicted state, and a predicted state can appear in different maximum combinations. For each combination, the Mahalanobis distances between the predicted state and any other hypothesized detections are computed, and the hypothesized detections with their Mahalanobis distances less than a threshold are used to update the corresponding predicted state as presented in the next section (Section 3.5). The hypothesized detections, which are not associated with any predicted state, are used to initialize new tracks in the same way as described in the initialization step. If no hypothesized detection is associated with the predicted state of a specific track, this track will be predicted to the next scs or stop at this scs based on its historical record, which will be detailed in Section 3.6.

### 3.5. Updating of Utility Tracks

After finishing the data association procedure described in Section 3.4, the EKF algorithm is employed to update the predicted tracks with their associated sensor hypothesized detections. As the data are captured by multiple sensors independently, when multiple hypothesized detections are associated with a specific track, this track can be updated by the hypothesized detections sequentially in any order. For a predicted track $X_{k|k-1}$ and an associated hypothesized detection $V_{k}^{i,m}$, the updating procedure is done in the standard way:

- measurement residual:  $d_{k}^{i,m}=V_{k}^{i,m}-H^{i}X_{k|k-1}$
- residual covariance:   $S_{k}^{i,m}=H^{i}P_{k|k-1}{\left(H^{i}\right)}^{T}+R_{k}^{i,m}$
- Kalman gain:      $G_{k}^{i,m}=P_{k|k-1}{\left(H^{i}\right)}^{T}{\left(S_{k}^{i,m}\right)}^{-1}$
- updated utility state:   $X_{k|k}=X_{k|k-1}+G_{k}^{i,m}d_{k}^{i,m}$
- update utility uncertainty: $P_{k|k}=\left(I-G_{k}^{i,m}H^{i}\right)P_{k|k-1}$

where $H^{i}$ is the observation model of sensor *i* and $R_{k}^{i,m}$ defines its Gaussian observation noise as $r_{k}^{i,m}∽N\left(0,R_{k}^{i,m}\right)$. The observation model function $H^{i}$ for sensor *i* used in this work is defined as: $H^{i}=\left[\begin{array}{cc} I_{5,5} & 0_{5,3} \end{array}\right]$, where $I_{5,5}$ is a 5×5 identity matrix and $0_{5,3}$ is a 5×3 zero matrix. For a sensor measurement only with 2D locations of the target, their observation model is a 4×8 matrix by deleting the third row of $H^{i}$.

### 3.6. Management of the Utility Tracks

In the course of utility tracks marching forward across a group of scss, an existing utility track with current state $X_{k}^{t}$ is predicted for the next scs and can be updated with associated sensor hypothesized detections. An updated utility track may be split into multiple tracks, and some tracks may be merged into a single track. Simultaneously, at an scs, some non-updated tracks may be pruned, and some new utility tracks may be initialized with new hypothesized detections. The different situations are described in the following bullets:

- split: if a predicted utility track can be associated with different groups of hypothesized detections, it is split into multiple tracks and updated with the corresponding hypothesized detection combinations, respectively;
- merge: if two utility tracks are updated with exactly the same hypothesized detections in *M* consecutive scss, they are merged as a single track. We tried a range of values of *M* from one to five in this work; the best result was obtained when *M* was set to three.
- prune: if a buried utility is detected on a scs and it extends forward to the following scss, the probability of this utility not being detected on several consecutive scss should be very low. Therefore, a variable $d_{no}^{t}$ is defined to record the accumulated non-updated utility distance among consecutive scss for a certain track. If the predicted state $X_{k|k-1}^{t}$ of the track on the *k*-th scan cross-section is not updated by any sensor hypothesized detection, the distance between the predicted location $X_{k|k-1}^{t}$ and the previous location $X_{k-1}^{t}$ is added onto $d_{no}^{t}$. When this accumulated distance exceeds a certain threshold (e.g., two metres), this utility track will be stopped. If any sensor hypothesized detection is associated with the track before $d_{no}^{t}$ reaching the threshold, $d_{no}^{t}$ will be reset to zero.
- new utility initialization: the hypothesized detections on $scs_{k}$ not associated with any predicted track are used to initialize new tracks in the same way as described in the initialization step (Section 3.2).

This procedure continues to the last scs to obtain all of the utility state estimates. For a certain track, its state estimates on all of the associated scan cross-sections are recorded. Among the state estimates, some of them are the results updated by hypothesized detections (called updated states), and others are just the predictions from the previous state (called prediction states). If the ratio of the number of updated states to the total number of state estimates is up to a threshold, this track is accepted as a utility segment; otherwise, it is believed to be a noise. The value of the threshold depends on the resolution of the scan lines and the quality of the sensor data. After this clean procedure, the accepted utilities are smoothed with a Rauch-Tung-Striebel (RTS) smoother [30,31,32].

#### Merging Utility Tracks Detected in Both Marching Directions

**Figure 4:**
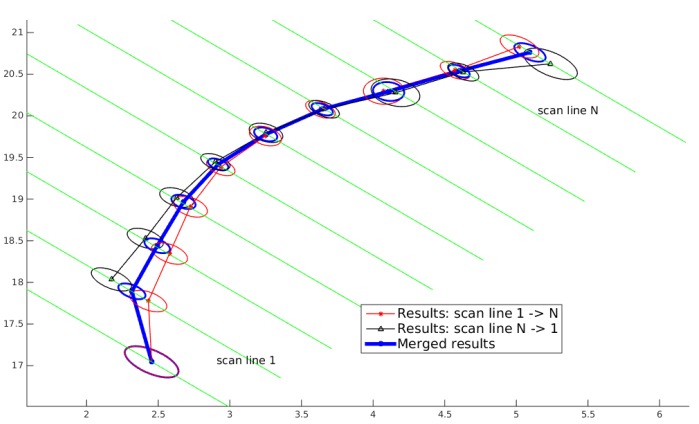
The utility segment after merging the corresponding utility segments from two directions. Uncertainties of utility states are shown by ellipse with 95% confidence level.

As explained above, once the proposed algorithm is applied in one order of the scan lines, say $sl_{1}$ to $sl_{N}$, the MCS algorithm is applied again from $sl_{N}$ to $sl_{1}$. By applying the algorithm twice, some segments misdetected in one direction might be detected in the other direction. By merging the results obtained in both directions, a more complete and more accurate final result can be achieved. An example is shown in Figure 4; it can be seen that one part of a segment, which is not detected when applying the MCS algorithm in one direction (black line), is detected when applying the algorithm in the other direction (red line). Meanwhile, after merging the results in two directions together, the uncertainties of the states of a detected utility are reduced (blue line). More examples are given in Section 5.

The following procedure is used for merging tracks detected by applying the MCS algorithm in both marching directions: (1) To determine if two tracks are from the same utility: for two tracks from different marching directions, the Mahalanobis distances of their states on the same scs are computed and compared. Let $N_{pairs}$ denote the number of utility state pairs with Mahalanobis distances less than a threshold and $N_{total}$ denote the total number of state pairs (states on the same scs) of two tracks. If the ratio $N_{pairs}/N_{total}$ is larger than a certain value, the two tracks are considered to be from the same utility. (2) to merge two tracks from the same utility: the states of the two tracks on the same scs are merged with Equations (4) and (5). If on a certain scs, only one track is detected there, the related state is used as the state of the final track.

The pseudo-code of the proposed MCS algorithm is given in Appendix B.

## 4. MCS Algorithm with Virtual Scan Lines

In some surveys, different sensors may not share common scan lines or only the coordinates of the hypothesized detections may be provided without any information of the scan lines. In this situation, to apply the previously-described algorithm, a group of virtual scan lines, which are parallel to each other, are automatically generated based on the provided hypothesized detections. Those hypothesized detections that are not on the generated scs in 3D space are projected onto their nearest virtual scss along a certain direction as described below. What is the orientation of the virtual scan lines, and what is the distance between two adjacent ones? These two questions are answered in the following sections.

### 4.1. Orientation of the Virtual Scan Lines

**Figure 5:**
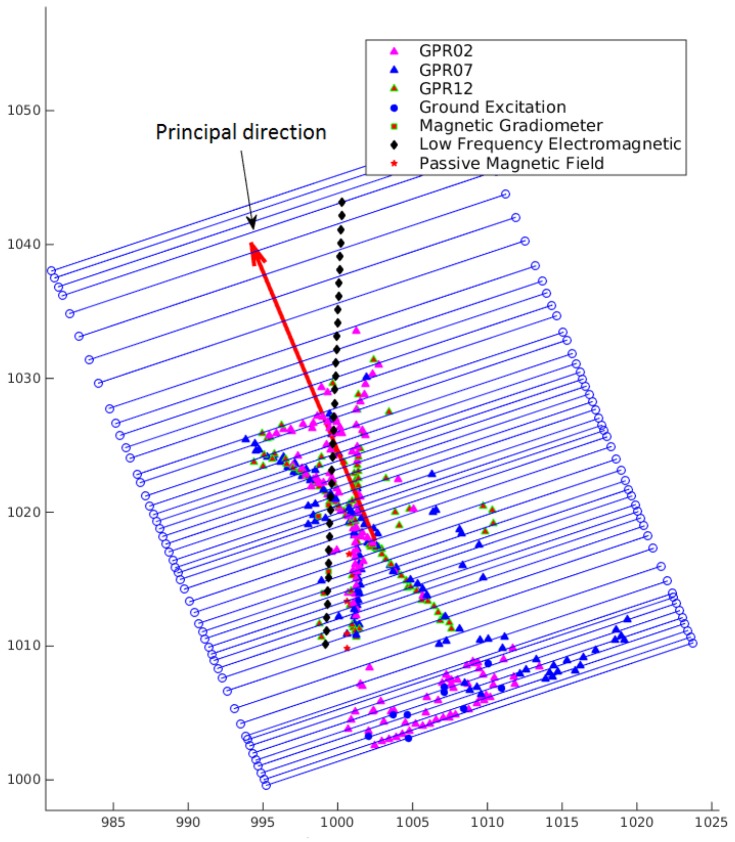
Virtual scan lines generated based on hypothesized detections from data of different surveys using five types of sensors. The main direction computed from the hypothesized detections is displayed as red bold arrow, and the virtual scan lines are shown as blue lines.

To decide the orientation of the virtual scan lines, the given hypothesized detections are projected onto the ground surface, and then, a PCA (Principal Component Analysis) [33] is applied to the projections of the hypothesized detections to find the first principal component direction, called the main direction. First, the direction perpendicular to the main direction is used as the direction of the virtual scan lines. An example is shown in Figure 5. To avoid missing the utilities with its longitudinal direction perpendicular or nearly perpendicular to the main direction, the proposed algorithm is repeated with the virtual scan lines parallel to the main direction, and then, the results from both directions are merged as described below in detail.

### 4.2. Adaptive Selection of Distance between Virtual Scan Lines

The distance between two adjacent scan lines can vary with respect to the local density of the hypothesized detections along the marching direction. In order to adaptively calculate the interval between virtual scan lines, the maximum distance between the projections of the hypothesized detections along the marching direction is first computed. Then, a group of grid lines with constant interval distance (e.g., 0.5 m) is generated along the marching direction. All the hypothesized detections are associated with their closest virtual scss, and an average number $A_{h}$ of hypothesized detections associated with an scs is computed based on this grid. If the number of hypothesized detections associated with a certain scs is greater than $2A_{h}$, then two new scss are added before and after this scs with a distance of a quarter of the original intervals they lie in to replace the original one. The number of hypothesized detections associated with these two new scss are computed. If any of them has hypothesized detections more than $2A_{h}$, then further dividing will be continued.

Since the scss are generated virtually, some hypothesized detections are not on the scs with which they are associated. In this situation the hypothesized detections are projected onto the virtual scss along a certain direction:

(a) if no utility track has been initialized prior to this scs, the associated hypothesized detections are projected onto the scs along the direction perpendicular to the scs. Then, the same initialization procedure is performed as described in Section 3.2;
(b) if some tracks have been initialized and predicted onto the current scs, the directions of the predicted tracks are used to project the hypothesized detections related to this scs: for each track, these hypothesized detections are projected along the predicted direction of the track, then the data association algorithm presented in Section 3.4 is applied to find the corresponding projected hypothesized detections of this utility track. This procedure is repeated for all of the predicted tracks;
(c) the hypothesized detections, which are not used to update any existing track, are projected along the direction perpendicular to the scs onto the related virtual scs and used to initialize new tracks.

Once the virtual scss are generated and with the proposed rule, the hypothesized detections are projected onto the current scs; the procedure is the same as that on a certain scs with actual scan lines.

When using virtual scss, if a utility is parallel or nearly parallel to the virtual scss, it may not be located by the above procedure. To avoid this, the above procedure is repeated twice with the virtual scan lines perpendicular and parallel to the main direction, respectively. In this way, the same utility segment might be detected in both directions. To merge the results obtained in both directions, the following rule is applied: if the angle *β* between a segment and the marching direction satisfies $\beta \leq 45^{\circ }$, this segment will be kept; otherwise, it will be rejected. The above rule is designed based on the fact that if a utility segment has a smaller angle with respect to one marching direction, it has a higher probability of being detected along this direction and in most cases with higher accuracy.

## 5. Experimental Results

In this section, the proposed MCS algorithm is tested on both synthetic and real data, and the experimental results are presented and analysed.

### 5.1. Synthetic Data

**Figure 6:**
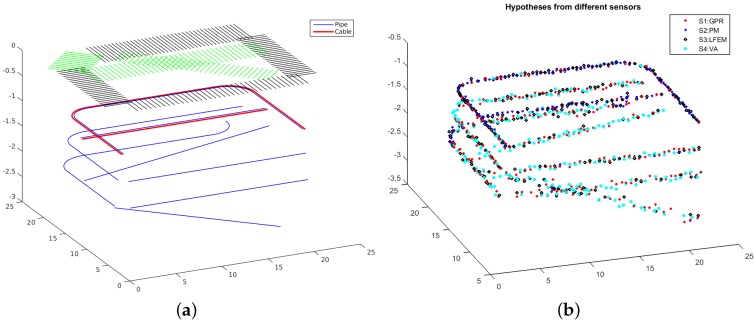
(**a**) A synthetic environment with several groups of scan lines on the ground surface and some utilities below the surface (three curved pipes, five straight pipes, one curved cable and one straight cable). The surveyed regions with green and black grids represent the grass covered-area and the tarmac area, respectively. A utility represented by a blue line is a pipe and otherwise a cable; (**b**) A group of synthetic hypothesized detections generated with the simulator with respect to the aforementioned synthetic environment for four sensors: GPR, PMF, Low Frequency Electromagnetic Fields (LFEM) and VA (best viewed in colour).

A simulator is designed to generate synthetic hypothesized detections of different sensors based on the location of buried utilities and the detection rates (the detection rate of a sensor with respect to a certain utility at certain depth in a specific medium is the probability of the utility being detected by the sensor when it is passing through the scan cross-section at that depth in the specified medium) of different sensors in different mediums (sensor data are not actually simulated). A synthetic environment including three curved pipes, five straight pipes, one curved cable and one straight cable is generated, as shown in Figure 6a. The pipes are assumed to be metal pipes, and the cables are assumed to be electrical cables. They are simulated at the depth of 1 m, 1.5 m and 2 m, respectively. Some of them are very close to each other, such as a curved pipe and a curved cable at the depth of 1 m and one straight cable and another straight pipe at the depth of 1.5 m. Seven groups of scan lines are simulated on a tarmac area and a grass area, with the black and green grids representing the scan lines on tarmac and on grass, respectively.

For a utility and a scan cross-section in the simulated environment, if the utility passes through the scan cross-section, the intersection point of them is computed by the simulator. Different levels of noise are added to the coordinates of the intersection point with respect to different sensors, different depths and different mediums. In addition, since different sensors have different detection rates in different mediums, there is a random process according to the related detection rate to decide if an intersection point with added noise is recorded as a hypothesized detection or not. For example, as pointed out in the previous sections that GPR works better on tarmac, this is because GPR is more accurate and has a higher detection rate on tarmac, say 0.8 at a depth of 1 m. If an intersection point is computed with respect to a survey with GPR on tarmac at a depth of 1 m, then this intersection point has a 0.8 probability to be recorded as a hypothesized detection. On the other hand, GPR works worse in grass areas, and given that the detection rate of GPR in wet grass area is 0.3 at a depth of 1 m, then a related intersection point at a depth of 1 m in the grass area has only a 0.3 probability to be recorded as a hypothesized detection. In our experiments, the detection rates are set inversely proportional to the depth: the constants of proportionality for different sensors in tarmac and grass-covered areas are respectively set as GPR, 0.8/0.3, PMF, 0.7/0.7, LFEM, 0.7/0.7, VA, 0.2/0.85. The simulated hypothesized detections from four sensors, GPR, PMF, LFEM and VA, are shown in Figure 6b.

**Figure 7:**
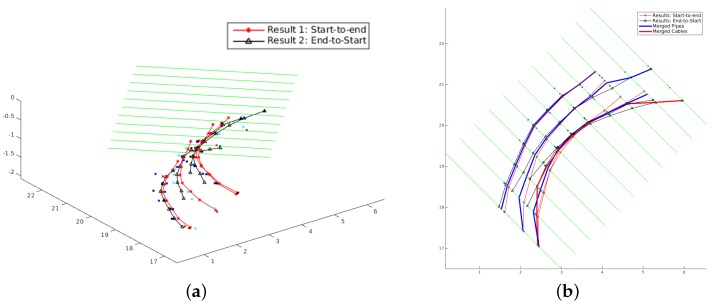
The results of the Marching-Cross-Section (MCS) algorithm by merging the corresponding results along two different marching directions. (**a**) 3D view of detected utility segments along two different marching directions; (**b**) 2D view of detected utility segments along two different marching directions and the final result after merging them together. After merging the results from two directions, the percentage of correctly detected utility segments increased from 84% and 87% to 95%. The missed 5% is due to no hypothesized detections generated in the corresponding area by any sensor.

The proposed MCS algorithm was applied to the seven groups of scan lines separately. As explained in Section 3.2, for each group of scan lines, the MCS algorithm is applied in two different directions. As seen in Figure 7a, four curved utility segments are detected respectively in each direction. By merging the corresponding tracks detected in both marching directions, the percentage of correctly-detected utility segments increases from 84% and 87% to 95%. The final utility segments are more accurate and more complete than using a single direction, as shown in Figure 7b.

**Figure 8:**
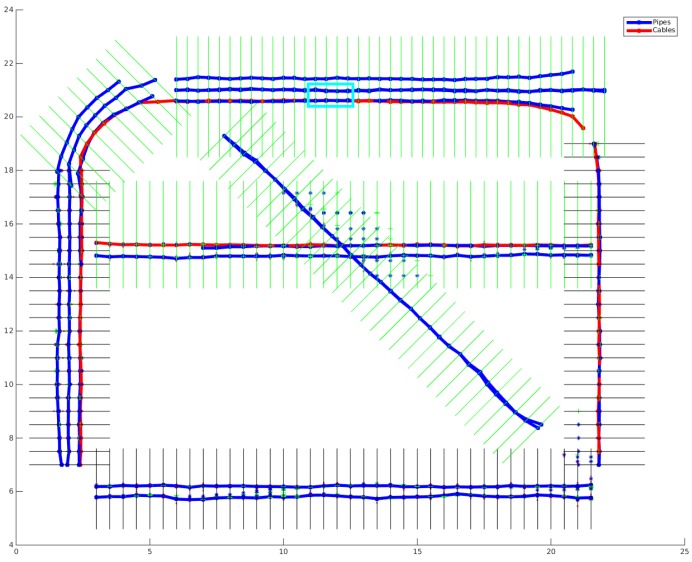
A top view of the straight and curved utilities located based on the simulated hypothesized detections. Located pipes and cables from the synthetic data are shown by blue lines and red lines, respectively.

For the whole seven groups of survey data, a top view of the located straight and curved utilities obtained with the proposed method is displayed in Figure 8. A utility segment detected between two scss is considered as a true positive detection if the distances from its two end points to a utility line given in the ground truth are both less than 10 cm. The accumulated length of correctly-detected utility segments and their average error of distance to the corresponding lines in the ground truth by using different groups of sensor data are shown in Table 1. It can be seen that the percentage of correctly located utility segments increases gradually by integrating the data from more and more sensors.

**Figure 9:**
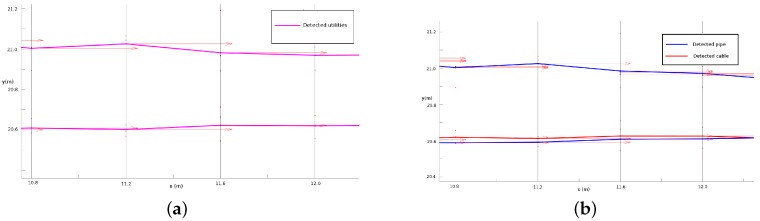
(**a**) Enlarged window of located utilities in the cyan window in Figure 8 when not using pp and pc; the types of located utilities cannot be estimated, and the cable segment and pipe segment that are close to each other cannot be separated. (**b**) The located utilities after using pp and pc, the cable and pipe, which are close to each other, can be separated successfully.

The detection rates pp and pc are important features of the MCS algorithm. They can help to estimate the possible type of a located utility and can help to separate a pipe from a cable when they are close to each other. An example is given in Figure 9a,b. It can be seen in Figure 9a that without using the information of pp and pc of the hypothesized detections, MCS merges the hypothesized detections from a cable and its neighbouring pipe into one utility, but by considering the pp and pc components, they can be separated successfully as two different utilities, as shown in Figure 9b. In addition, pp and pc can help to indicate the possible type of a located utility. If the value of pp of a located utility is higher than its pc, the located utility is considered to be a pipe and shown by a blue line; otherwise, it is believed to be a cable and shown by a red line. As seen in Figure 9b, both the location and the type of the utilities match correctly with the ground truth. The values of pp and pc for different sensors used in this work are given in Table 2, which are assigned according to their operational capabilities summarized in [34].

In order to test the algorithm presented in Section 4, we assume that the information of the seven groups of scan lines is not provided, then a group of virtual scan lines is automatically generated based on all of the hypothesized detections using the algorithm presented in Section 4. With this group of virtual scan lines, all of the hypothesized detections from different surveys are processed in the same framework. As can be seen in Table 1, the utility location results are very close to the results of using actual scan lines. The MCS algorithm with virtual scan lines is especially useful when a large quantity of utility hypothesized detections from different surveyed data sources are provided.

### 5.2. Real Data

#### 5.2.1. Survey Site

The Mapping the Underworld (MTU, http://www.mappingtheunderworld.ac.uk/) project surveyed the Glen Eyre halls of residence at Southampton University, UK, to test the efficiency of the proposed algorithm for integrating different subsurface utility locating techniques. The survey was carried out in a car park, approximately 30 m by 20 m. The site is mainly tarmac with a small grass-covered area. Three control points were set up to generate a coordinate system with a Leica total station and all of the sensor data were calibrated to this coordinate system. Two GPR sensors, a PMF sensor, a MG sensor, a LFEM sensor and a VA sensor for ground excitation were applied to capture data on-site in 2015.

The GPR data were captured in almost the whole area along several groups of scan lines in different directions. For each group of scan lines, the scan lines are parallel to each other approximately, and the distance between adjacent scan lines is about 0.5 m. The PMF sensor was performed in an area of about 8 m × 5 m with the length of a scan line about 5 m and the distance between two adjacent scan lines about 1 m. The survey of MG covered a region of about 10 m × 20 m on tarmac with some overlap with the region surveyed by PMF and LFEM. The LFEM survey covered a large portion of the tarmac area, and GPS, a laser scanner and Odometry were used to provide the positional information for the LFEM hypothesized detections. The survey of the VA of ground excitation covered a portion of the grass area. Seven geophones were deployed along the scan lines with the distance between two adjacent geophones about 1 m.

#### 5.2.2. Ground Truth

**Figure 10:**
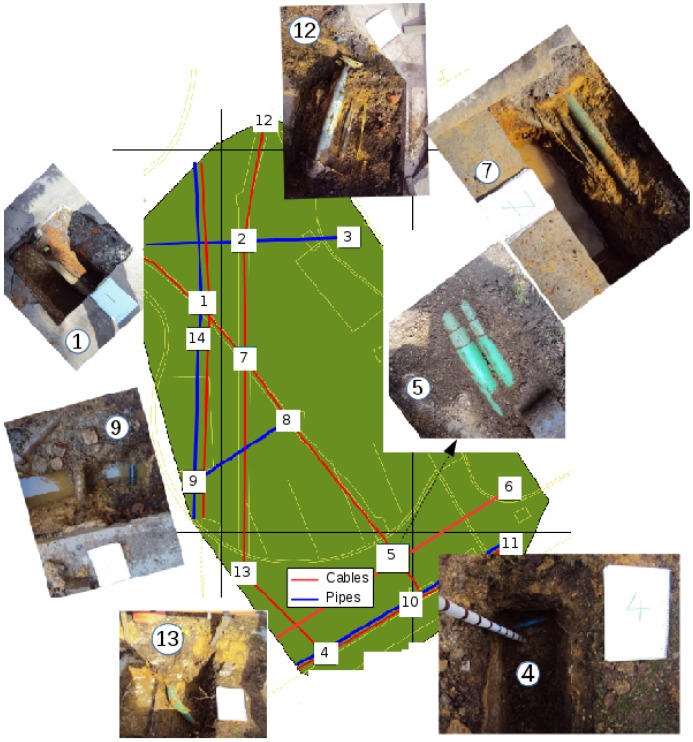
Test site with pictures of dug pits and the ground truth of buried utilities including cables (red lines) and main pipes (blue lines) provided by excavations after all sensor acquisitions.

In March 2016, 12 of 14 picked trial pits were successfully excavated by a professional excavating company to a depth of around 1 m to obtain ground truth of buried utilities in the test site (thus, deep utilities may not have been discovered). Multiple cables and pipes were found in different pits as can be seen in Figure 10. If similar utilities with similar orientations and depths are found in neighbouring pits, they are assumed to be from the same utility and connected linearly as the ground truth. The same as with the synthetic data, for a detected utility segment between two adjacent scan cross-sections, if the distances of its two ends to any utility in the ground truth are both less than 0.3 m, it is recorded as being located correctly.

#### 5.2.3. Experiment I: MCS Algorithm Applied on Three Groups of GPR Data Sharing Common Scan Lines

**Figure 11:**
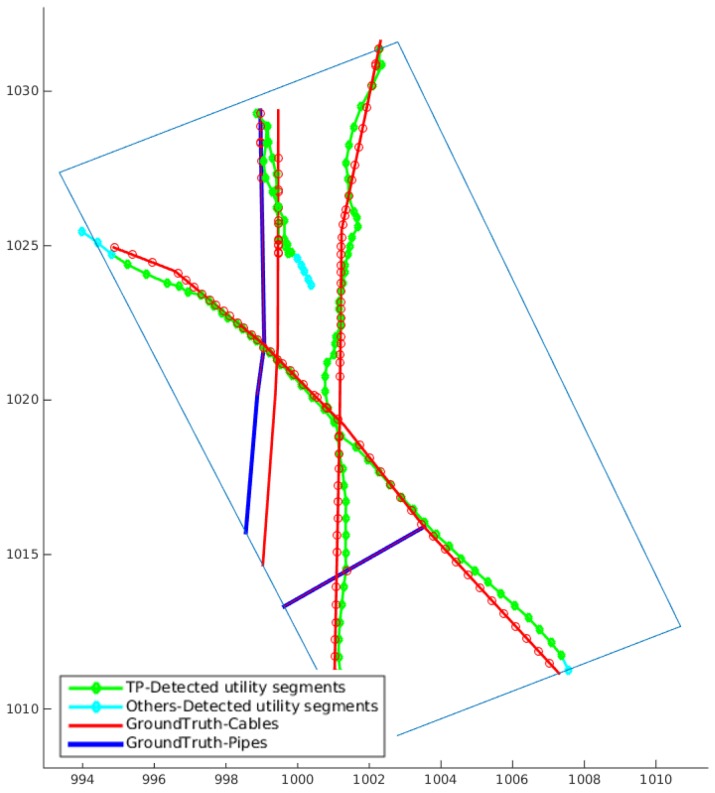
Experimental results of the MCS algorithm with three groups of GPR data sharing common scan lines in the Glen Eyre test site. The ground truth in the surveyed area is shown by blue and red solid lines; correctly located utility segments (TP: true positive) are marked by green dotted lines with the red circles on the ground truth lines the corresponding closest points of the ground truth; and false positive/unknown utility segments are marked by cyan dotted lines (best viewed in colour).

Three groups of GPR data, which share common scan lines, were used to test the MCS algorithm presented in Section 3. As shown in Figure 11, five utility tracks were retained. The detected utility segments are compared with the ground truth, and the correctly detected utility segments are marked by green dotted lines. By using the three subsets of GPR data, 60.7% of the utilities in the surveyed area were detected.

**Figure 12:**
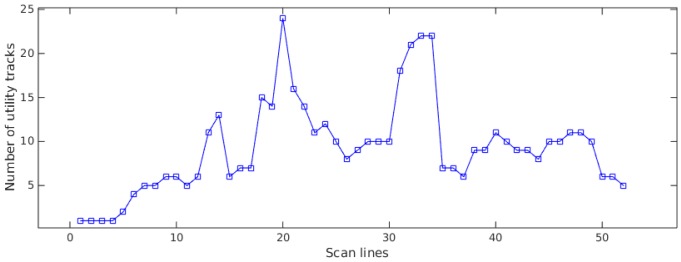
Number of utility tracks on different scan cross-sections (scss).

The number of utility tracks at different scss is displayed in Figure 12; the MCS algorithm can track multiple utilities and the number of utility tracks varies from step to step because of different numbers and/or different distributions of hypothesized detections extracted on different scss. There are two main peaks in Figure 12: the first one is because of the intersection of two long utilities, and then, the number of tracks drops drastically in the following steps thanks to the tracks merging strategy; the second peak is because more hypothesized detections were extracted on the corresponding scan cross-section.

#### 5.2.4. Experiment 2: MCS Algorithm with Virtual Scan Lines for Multiple Groups of Sensor Data

For all of the data captured from the five types of sensors in the test site, since only a small number of groups among them share common scan lines and some of them only provide the locations of hypothesized detections, in order to use all of these data in a common framework, the algorithm described in Section 4 is applied to generate a group of virtual scan lines. To evaluate the contribution of using multiple sensors, MCS is applied on different combinations of sensors. Since GPR data cover a larger proportion of the surveyed area than other sensors, MCS is applied on the GPR data at first to generate a baseline. Then, MCS is applied by gradually adding other sensor data into the experiments to evaluate the contribution of different sensors.

**Figure 13:**
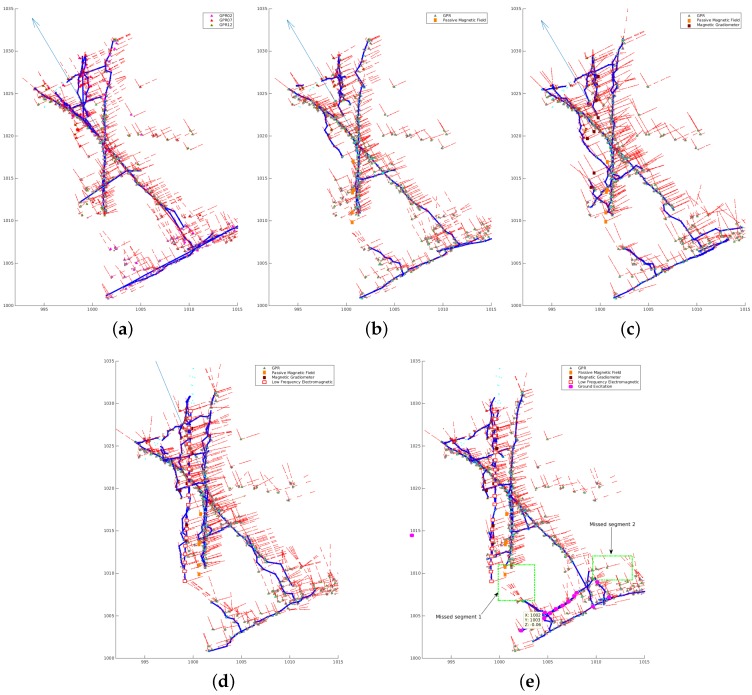
Experimental results of the MCS algorithm with virtual scan lines on real data captured in the Glen Eyre survey. (**a**) GPR; (**b**) GPR + PMF; (**c**) GPR + PMF + MG; (**d**) GPR + PMF + MG + LFEM; (**e**) GPR + PMF + MG + LFEM + VA. Hypothesized detections from different sensors are marked by different symbols, as shown in the legend; projections of the hypothesized detections on the corresponding virtual scan lines are marked by cyan dots; the predicted utility states at different locations are marked by red line arrows; and the located buried utilities are marked by bold blue lines.

The experimental results with respect to different combinations of sensors are shown in Table 3, and the location results with respect to certain combinations of data groups from the MCS algorithm are displayed in Figure 13.

Since in the surveys, different sensors covered different portions of the whole surveyed area and with some overlaps of the areas they covered, a correctly located segment may be based on the data from multiple sensors, and different sensors may detect different parts of the same utility or different utilities, resulting in a more complete utility location result. As seen in Table 3 and Figure 13, with more sensor data fed into the MCS algorithm, the length of correctly located utilities is longer and the location result is more accurate. By integrating all sensor data together, 92% of the utilities are located correctly. For the remaining 8% of the utilities, the related areas are not covered by any sensor, as pointed out in the two green rectangle areas in Figure 13e.

## 6. Conclusions

In this paper, a novel algorithm MCS for automatically locating buried utility segments by fusing data from multiple sensors is introduced. By discretizing the 3D space with scan cross-sections based on actual survey scan lines or automatically generated virtual scan lines, the MCS algorithm marches from a scan cross-section to the next one by predicting the states of detected utilities on the next cross-section. The predicted states of utilities can be integrated with hypothesized detections from multiple sensors to obtain better estimations. The proposed marching algorithm can detect multiple straight or curved buried utility segments simultaneously. The novel idea of formulating pp and pc into the algorithm can help to separate pipe and cable close to each other. Based on the analysis of the experimental results on both synthetic data and real data, the novel formulation proposed for the EKF and the rules proposed for associating predicted utilities with the hypothesized detections from multiple sensors on a scan cross-section in the MCS algorithm are shown to work well for buried utility location. Given hypothesized detections from multiple sensors in a surveyed area, the utilities under the surveyed area can be correctly located with high recall and precision by the proposed MCS algorithm in a fully automatic manner. The proposed method is a greedy method and, thus, might not find the global optimum, though this is mitigated by combining the two marching directions. Future work might address the development of an algorithm, which searches for a global optimum, though we note good results are already obtained with the present algorithm.

## Figures and Tables

**Table 1:** Comparison of the utility location results using different sensor combinations. Both the results of MCS with actual survey scan lines (sls) and virtual scan lines are presented. RCD is the rate of correctly detected utility length compared with the total utility length in the ground truth, RCD∈[0,1]; $\bar{E}$ (m) is the average distance error of the detected utilities. In this table, S1, S2, S3 and S4 represent GPR, PMF, LFEM and VA, respectively.

Sensors	MCS (Actual *sl*s)				MCS (Virtual *sl*s)	
	Tarmac Area		Grass Area		Whole Area	
	RCD	$\bar{E}$	RCD	$\bar{E}$	RCD	$\bar{E}$
S1	0.57	0.12	0.84	0.06	0.58	0.08
S2	0.38	0.05	0.27	0.03	0.27	0.04
S3	0.66	0.07	0.60	0.07	0.40	0.07
S4	0.53	0.05	0.53	0.07	0.51	0.08
S1-2	0.81	0.06	0.89	0.05	0.74	0.07
S1-3	0.81	0.05	0.82	0.04	0.85	0.07
S1-4	0.54	0.05	0.88	0.04	0.69	0.07
S2-3	0.65	0.04	0.61	0.05	0.50	0.06
S2-4	0.91	0.05	0.81	0.05	0.78	0.07
S3-4	0.91	0.05	0.89	0.04	0.85	0.07
S1-2-3	0.84	0.05	0.89	0.04	0.98	0.07
S1-2-4	0.92	0.05	0.93	0.04	0.90	0.07
S1-3-4	0.93	0.05	0.93	0.03	0.89	0.06
S2-3-4	0.93	0.05	0.89	0.04	0.90	0.07
S1-2-3-4	0.94	0.04	0.93	0.03	0.93	0.04

**Table 2:** The probability of an extracted hypothesized detection being from a pipe or a cable for different sensors: pp, the probability of a hypothesized detection to be a pipe; pc, the probability of a hypothesized detection to be a cable; po, the probability of a hypothesized detection to be other types of objects.

Sensor	pp: Pipes	pc: Cables	po: Others
GPR	0.5	0.35	0.15
PMF	0.05	0.9	0.05
MG	0.05	0.9	0.05
LFEM	0.45	0.45	0.1
VA	0.85	0.1	0.05

**Table 3:** Experimental results of MCS with virtual scan lines on the data of the Glen Eyre site survey: RCD is the rate of correctly located utility length compared with the ground truth, RCD∈[0,1]; $\bar{E}$ (m) is the average distance error of the located utility segments.

Sensors/Results	MCS Algorithm	
	RCD	$\bar{E}\left(m\right)$
GPR	0.64	0.25
GPR + PMF	0.68	0.23
GPR + PMF + MG	0.71	0.23
GPR + PMF + MG + LFEM	0.85	0.20
GPR + PMF + MG + LFEM + VA	0.92	0.20

## Appendix A. Derivation of Equation (7)

With respect to a utility with state vector as $X_{k-1}={\left(x_{k-1},y_{k-1},z_{k-1},pp_{k-1},pc_{k-1},dx_{k-1},dy_{k-1},dz_{k-1}\right)}^{T}$ on scan cross-section $scs_{k-1}$ in Figure 2, to compute the prediction of this utility on $scs_{k}$, the length of the utility segment $l_{k}$ between $scs_{k-1}$ and $scs_{k}$ needs to be computed. Let *l* be a real number, then the equation of the line containing the utility segment can be represented as:

(A1) $$\left\{\begin{array}{ccc} x-x_{k-1} & = & l\cdot dx_{k-1},\\ y-y_{k-1} & = & l\cdot dy_{k-1},\\ z-z_{k-1} & = & l\cdot zx_{k-1}, \end{array}\right.$$

Since the start point of the *k*-th scan line is $\left(x_{k}^{s},y_{k}^{s},z_{k}^{s}\right)$ and the normal vector of $sc_{k}$ is $\left(a_{k},b_{k},0\right)$, the equation of the plane containing $sc_{k}$ is:

(A2) $$a_{k}\left(x-x_{k}^{s}\right)+b_{k}\left(y-y_{k}^{s}\right)=0$$

Plug Equation (A1) into Equation (A2), we obtain:

(A3) $$l=\frac{a_{k}\cdot \left(x_{k}^{s}-x_{k-1}\right)+b_{k}\cdot \left(y_{k}^{s}-y_{k-1}\right)}{a_{k}\cdot dx_{k-1}+b_{k}\cdot dy_{k-1}}$$

In this situation, the meaning of *l* is exactly the same as $l_{k}$; this proves Equation (7).

## Appendix B.

**Algorithm B1:** Pseudo-Code of the Proposed MCS Algorithm

## References

[1] Burtwell M., Faraghe E.R., Neville D., Overton C., Roger C., Woodward T. (2004). Locating Underground Plant and Equipment Proposals for a Research Programme.

[2] Metje N., Atkins P., Brennan M., Chapman D., Lim H., Machell J., Muggleton J., Pennock S., Ratcliffe J., Redfern M. (2007). Mapping the Underworld-State-of-the-art review. Tunn. Undergr. Space Technol..

[3] Muggleton J., Brennan M., Gao Y. (2011). Determining the location of buried plastic water pipes from measurements of ground surface vibration. J. Appl. Geophys..

[4] Muggleton J., Papandreou B. (2014). A shear wave ground surface vibration technique for the detection of buried pipes. J. Appl. Geophys..

[5] Muggleton J., Brennan M., Rogers C. (2014). Point vibration measurements for the detection of shallow-buried objects. Tunn. Undergr. Space Technol..

[6] Goddard K.F., Wang P., Lewin P.L., Swingler S.G. (2012). Detection and location of underground cables using magnetic field measurements. Meas. Sci. Technol..

[7] Foo K., Atkins P., Thomas A., Rogers C. Capacitive-Coupled Electric-Field Sensing for Urban Sub-Surface Mapping: Motivations and Practical Challenges. Proceedings of the International Conference on Frontiers in Shallow Subsurface Technology.

[8] Mertens L., Persico R., Matera L., Lambot S. (2016). Automated detection of reflection hyperbolas in complex GPR images with no a priori knowledge on the medium. IEEE Trans. Geosci. Remote Sens..

[9] Chen H., Cohn A.G. Probabilistic conic mixture model and its applications to mining spatial ground penetrating radar data. Proceedings of the Workshops in SIAM Conference on Data Mining (SDM10).

[10] Metje N., Chapman D.N., Cheneler D., Ward M., Thomas A.M. (2011). Smart pipes-instrumented water pipes, can this be made a reality?. Sensors.

[11] Royal A.C.D., Atkins P.R., Brennan M.J., Chapman D.N., Chen H., Cohn A.G., Foo K.Y., Goddard K.F., Hayes R., Hao T. (2011). Site assessment of multiple-sensor approaches for buried utility detection. Int. J. Geophys..

[12] Dutta R., Cohn A.G., Muggleton J.M. (2013). 3D mapping of buried underworld infrastructure using dynamic Bayesian network based multi-sensory image data fusion. J. Appl. Geophys..

[13] Li S., Cai H., Kamat V.R. (2015). Uncertainty-aware geospatial system for mapping and visualizing underground utilities. Autom. Constr..

[14] Chen H., Cohn A.G. Buried utility pipeline mapping based on multiple spatial data sources: a Bayesian data fusion approach. Proceedings of the Twenty-Second International Joint Conference on Artificial Intelligence.

[15] Maybeck P. (1982). Stochastic Models, Estimation and Control.

[16] Jazwinski A.H. (1970). Stochastic Processes and Filtering Theory.

[17] Bar-Shalom Y., Li X.R., Kirubarajan T. (2001). Estimation with Applications to Tracking and Navigation.

[18] Sarkka S. (2013). Bayesian Filtering and Smoothing.

[19] Kim C., Li F., Ciptadi A., Rehg J.M. Multiple hypothesis tracking revisited. Proceedings of the IEEE International Conference on Computer Vision.

[20] Dou Q., Wei L., Magee D., Cohn A. (2016). Real Time Hyperbolae Recognition and Fitting in GPR Data. IEEE Trans. Geosci. Remote Sens..

[21] Wang P., Goddard K., Lewin P., Swingler S. Electromagnetic Field Application to Underground Power Cable Detection. Proceeding of the International Symposium on High Voltage Engineering.

[22] Muggleton J., Papandreou B.D., Brennan M. Detection of Buried Pipes using a Shear Wave Ground Surface Vibration Technique. Proceeding of the 19th International Congress on Sound and Vibration.

[23] Zhu S., Wang J., Su Y., Sato M. A circular survey for 3D ground penetrating radar to map hidden cylinders. Proceeding of the 7th International Workshop on Advanced Ground Penetrating Radar (IWAGPR).

[24] Zoubir A.M., Chant I.J., Brown C.L., Barkat B., Abeynayake C. (2002). Signal processing techniques for landmine detection using impulse ground penetrating radar. IEEE Sens. J..

[25] Swingler S., Wang P., Hao T., Chapman D., Curioni G., Foo K., Atkins P., CDF R. Flangeless open-ended coaxial probes with curved surface. Proceeding of the IEEE International Geoscience and Remote Sensing Symposium.

[26] Wang P., Goddard K.F., Lewin P.L., Swingler S.G., Atkins P.R., Foo K. Magnetic Field Measurement to Detect and Locate Underground Power Cable. Proceeding of the International Conference on Pipelines and Trenchless Technology.

[27] McKenna S.P., Parkman K.B., Perren L.J., McKenna J.R. (2013). Automatic Detection of a Subsurface Wire Using an Electromagnetic Gradiometer. IEEE Trans. Geosci. Remote Sens..

[28] Opfer J., Yeo Y., Pierce J., Rorden L. (1974). A superconducting second-derivative gradiometer. IEEE Trans. Magn..

[29] Manning C.D., Raghavan P., Schütze H. (2008). Introduction to Information Retrieval.

[30] Cox H. (1964). On the estimation of state variables and parameters for noisy dynamic systems. IEEE Trans. Autom. Control.

[31] Rauch H.E., Tung F., Striebel C.T. (1965). Maximum likelihood estimates of linear dynamic systems. AIAA J..

[32] Sage A.P., Melsa J.L. (1971). Estimation Theory with Applications to Communications and Control.

[33] Jolliffe I. (1986). Principal Component Analysis.

[34] Mapping the Underworld Brochure. http://www.mappingtheunderworld.ac.uk/MTU%20Brochure%20Final%20Version.pdf.

